# Long-Term Effects of Allergen Sensitization and Exposure in Adult Asthma: *A Prospective Study*

**DOI:** 10.1097/WOX.0b013e3181a45f96

**Published:** 2009-05-15

**Authors:** Stephen J Fowler, Stephen J Langley, Nicholas J Truman, Ashley Woodcock, Angela Simpson, Adnan Custovic

**Affiliations:** 1 University of Manchester, Manchester, UK; 2 Lancashire Teaching Hospitals NHS Trust, Preston, UK

**Keywords:** asthma, allergens, environmental exposure, longitudinal studies

## Abstract

**Background**

We investigated the effects of sensitization and exposure to common domestic allergens on longitudinal changes in lung function and bronchial hyperresponsiveness.

**Methods**

Subjects attended 2 visits that were 4 years apart. Skin prick testing was performed and household dust samples were collected for quantification of mite, dog, and cat allergens at baseline. Measurements of lung function, exhaled nitric oxide, and bronchial hyperresponsiveness were completed at both visits.

**Results**

Dust samples were collected in 165 of the 200 subjects completing both visits. Mean length of follow-up was 47 months. Bronchial hyperresponsiveness, measured at both visits in 86 subjects, deteriorated in those exposed to high mite allergen levels compared with those not exposed [mean (95% CI) doubling dose change PD_20 _= -0.44 (-1.07 to 0.19) vs 0.82 (0.27 to 1.36)], but improved in those exposed to high dog allergen levels compared with those not exposed [1.10 (0.33 to 1.86) vs 0.10 (-0.39 to 0.58)]. The associations were significant in the multivariate models. Cat allergen exposure was not associated with any changes in lung function, exhaled nitric oxide, or bronchial hyperresponsiveness.

**Conclusions**

In a 4-year prospective cohort of persons with asthma, exposure to high levels of dust mite allergens at baseline was associated with a subsequent increase in bronchial hyperresponsiveness.

## Introduction

Progressive deterioration in symptoms and an accelerated decline in lung function are features of the natural history of disease in a proportion of patients with asthma [1]. Identifying factors associated with this decline would be an important step in the development of strategies aimed at preventing long-term deterioration, compared with current recommendations that principally address short-term symptom control [2].

Among various factors associated with the decline over time, the clearest evidence to date has linked cigarette smoking to a loss of lung function [3,4]. Although patients with bronchial hyperresponsiveness (BHR) are less likely to develop irreversible airflow obstruction, the presence of BHR has been associated with an accelerated decline in lung function [5,6]. There is no clear link between atopic sensitization and decline in lung function in asthma. However, the absolute level of specific IgE has been related to the development of wheezing in children and a number of positive skin tests to increasing BHR in adults [7,8].

The ASMAL (*AS*sessment of *M*anchester *A*sthmatics *L*ongitudinally) cohort was set up to investigate factors associated with asthma control and lung function [9-11]. Baseline cross-sectional data suggested that, for sensitized individuals, domestic exposure to high levels of sensitizing allergens is associated with poorer lung function [11]. Surprisingly, even among nonsensitized persons with asthma, high levels of exposure to dust mite or dog allergen were associated with increased BHR [10]. On the basis of these findings, we hypothesized that allergen sensitization and high allergen exposure would be associated with a decline in lung function and increased BHR and airway inflammation over time. Thus, within the ASMAL cohort, we aimed to investigate the effects of sensitization and domestic exposure to common inhalant allergens (dust mite, cat, and dog) on longitudinal changes in lung function and BHR over a 4-year period.

## Methods

### Participants

We recruited subjects with physician-diagnosed asthma (asthma symptoms in the previous 12 months, treatment with a minimum of a short-acting bronchodilator, and no exacerbations for 4 weeks before recruitment) as previously described [10,11]. The study was approved by the local research ethics committee; subjects provided written informed consent.

Participants attended 2 visits 4 years apart. Previsit medication was withheld as follows: short-acting bronchodilators, 6 hours; long-acting bronchodilators, theophyllines, and leukotriene receptor antagonists, 12 hours; antihistamines, 48 hours.

### Procedures

#### Exhaled Nitric Oxide

Exhaled nitric oxide (eNO) was measured using a chemiluminescence analyzer (LR2000; Logan Research, Rochester, UK) according to published guidelines [12]. Subjects exhaled from inspiratory capacity to residual volume at a controlled rate (6 L/min) into a wide-bore minimal-resistance Teflon tube. Response time (10%-90%) was < 0.6 seconds and the sampling rate was 250 L/min; the final value was recorded as the mean of 2 maneuvers.

#### Spirometry

Spirometry was performed according to ATS guidelines [13] (Microlab 3000; MicroMedical, Rochester, UK).

#### Bronchial Challenge

Bronchial challenge was performed at baseline using either methacholine or histamine. Supply problems precluded the use of the same challenge in all subjects, but previous studies have demonstrated dose equivalence;[14,15] analysis of our data found no evidence of differences between the challenges and no bias in terms of outcomes [10,11]. At the follow-up visit, all subjects had histamine challenge. For either challenge, a modified Yan technique was used [16] [Koko DigiDoser dosimeter (PDS Research, Louisville, Colo) and "646" nebulizer pots (DeVilbiss, Somerset, Pa)]. Baseline FEV_1 _(FEV_1_, forced expiratory volume in 1 second) measurement (best of 3) was performed 1 minute after inhalation of isotonic saline, then cumulative doubling doses of the challenge solution were administered, and FEV_1 _was measured 1 minute after each dose. When the FEV_1 _had fallen by 20% or more, the challenge was terminated and PD_20 _calculated by linear interpolation. Where the FEV_1 _did not fall by 20% despite the maximum dose of challenge solution, aPD_20 _of 12 mg was assigned (1 doubling dose above the maximum given).

#### Sensitization

Sensitization was ascertained by skin prick testing (mite, cat, and dog; Bayer, Elkhart, Ind), and sensitization is defined as a mean wheal diameter of at least 3 mm greater than the negative control.

#### Allergen Exposure

Allergen exposure was assessed in dust samples collected from homes by vacuuming a 1-m^2 ^area of living room carpet and mattress for 2 minutes. Mite, cat, and dog allergen levels were measured using monoclonal antibody-based enzyme-linked immunosorbent assays [17-19].

### Statistical Analysis

Statistical analysis was performed using SPSS 12.0 for Windows. The primary end point was change in lung function (FEV_1 _and BHR) over time in relation to sensitization status and allergen exposure; 250-mL change in FEV_1 _and 1 doubling dose change in PD_20 _were considered relevant. On the basis of the data at the baseline visit, a sample size of 200 provided 80% power to detect a 250-mL change in FEV_1 _and a sample size of 106 to detect a 1 doubling dose change in PD_20 _(*α *error = 0.05). Allergen levels, eNO, and PD_20 _were normalized using log-transformation. Between-group differences for nominal variables were investigated using the *χ*^2 ^test. Lung function variables at 2 visits were compared using the paired *t *test, both overall and for subgroups. Change in lung function over time was calculated and between-group comparisons were made initially by independent *t *tests. Further comparisons were performed using analysis of variance (ANOVA), with possible confounders included in the multivariate model (*P *< 0.5). ANOVA was employed to investigate possible interaction between exposure and sensitization.

## Results

### Participants

Two hundred of the original cohort of 399 persons with asthma attended the follow-up visit, at mean 47 months (range of 25-68 months) after the baseline assessment. Subjects attending for follow-up tended to be older (44 vs 35 years old, *P *= 0.001) with a longer duration of asthma (23 vs 17 years, *P *= 0.001), were more likely treated with inhaled corticosteroids (72% vs 58%, *P *= 0.005), and had worse lung function (FEV_1 _= 2.50 L vs 3.05 L, *P *< 0.001) compared with those who did not attend (Table 1).

**Table 1:** Characteristics of Subgroups Included in the Analysis*

	Attended Both Visits		Allergen Samples Collected		Bronchial Challenge at Both Visits	
	
	Yes	No	Yes	No	Yes	No
	N = 200	N = 199	N = 165	N = 35	N = 86	N = 79
Age (years)	44	35†	45	41	41	50†
		(n = 195)				
Sex (% males)	40%	44%	39%	43%	31%	48%†
Years with asthma	23	17†	23	23	20	25†
	(n = 179)	(n = 132)	(n = 150)	(n = 31)	(n = 72)	(n = 78)
% on ICS treatment	72%	58%†	73%	66%	60%	86%†
	(n = 199)			(n = 34)		
ICS dose (*μ*g)	967	1034	948	1064	830	1042
	(n = 137)	(n = 101)	(n = 115)	(n = 22)	(n = 51)	(n = 64)
Smoking status (%)						
Never	61%	61%	59%	71%	65%	52%
Ex	28%	23%	30%	17%	27%	33%
Current	12%	16%	12%	11%	8%	15%
Spirometry						
FEV_1 _(l)	2.50	3.05†	2.51	2.49	2.87	2.11†
	(n = 197)	(n = 199)	(n = 164)	(n = 33)		(n = 78)
FVC (l)	3.48	3.80†	3.48	3.46	3.73	3.27†
	(n = 166)	(n = 110)	(n = 137)	(n = 29)	(n = 63)	(n = 74)
Reversibility (%)	14%	13%	14%	14%	8%	18%†
	(n = 124)	(n = 73)	(n = 106)	(n = 18)	(n = 40)	(n = 66)
PD_20 _GM (*μ*g)	0.412	0.538	0.422	0.398	0.394	0.590
	(n = 118)	(n = 146)	(n = 103)	(n = 15)		(n = 17)
eNO GM (ppb)	11.09	9.76	11.04	11.41	10.77	11.35
	(n = 173)	(n = 160)	(n = 149)	(n = 24)	(n = 80)	(n = 69)
Atopy (%)	84%	83%	82%	94%	85%	80%
	(n = 195)	(n = 195)	(n = 164)	(n = 31)	(n = 85)	(n = 79)

*Where data not available for all subjects in group, the number included in the analysis is shown in parentheses.

†*P *< 0.05 "no" vs "yes".

ICS, inhaled corticosteroids.

Of 200 subjects with a longitudinal data collection, 165 had domestic allergen levels determined at visit 1; there were no differences in any of the measurements between subjects with or without allergen exposure assessment. Of these 165, 103 had a bronchial challenge at visit 1 (reasons for not being tested: 31/62 had FEV_1 _< 60%; the remaining subjects declined the test) and 110 at visit 2 (22/55 had FEV_1 _< 60%; the rest declined the test). The 86 subjects who had bronchial challenge at both visits were less often treated with inhaled corticosteroids (60% vs 86%, *P *< 0.001), had better lung function (FEV_1 _= 2.87 L vs 2.11 L, *P *< 0.001; FVC = 3.73 L vs 3.27 L, *P *< 0.001; FVC, forced vital capacity), and were less likely to be male (31% vs 48%, *P *= 0.04). Characteristics of the study population are presented in Table 2.

**Table 2:** Baseline Characteristics of Participants With Allergen Samples Collected

	n		
Age (years)	165	Mean 45	
		Range 10 to 67	
Sex	165	39% male	
Years with asthma	150	Mean 23	
		Range 1 to 59	
Age diagnosed with asthma (years)	150	Mean 23	
		Range 0 to 58	
ICS treatment	165	73%	
ICS dose (BDP dose equivalent, *μ*g)	115	Mean 948	
		Median 800	
		Range 100 to 4000	
Smokers	165	Never	59%
		Ex	30%
		Current	12%
Lung function			
FEV_1 _(l)	164	Mean 2.51	
		Range 0.71 to 4.91	
FVC (l)	137	Mean 3.48	
		Range 1.41 to 7.13	
PD_20 _(mg)	103	GM 0.422	
		95% CI 0.303 to	
		0.585	
Classification of BHR	103	None or borderline	10%
		(PD_20 _> 4 mg)	
		Mild	20%
		(PD_20 _> 1-4 mg)	
		Moderate to severe	70%
		(PD_20 _≤ 1 mg)	
Exhaled nitric oxide (ppb)	149	GM 11.04	
		95% CI 9.66 to 12.61	
Atopic (SPT done in 164 subjects)		82%	
Sensitized to:			
Dust mite			61%
Cat			49%
Dog			43%

BDP, beclomethasone dipropionate; SPT, skin prick testing.

### Change in Lung Function, eNO, and BHR Over Time

The change in lung function, eNO, and BHR between 2 visits in the whole population and among sensitized and nonsensitized subjects are presented in Table 3. In all subjects, FEV_1 _(% predicted value) increased over time from 82.0 to 84.2% predicted [95% CI for difference 0.2-4.1% predicted, *P *= 0.03], although the absolute FEV_1 _did not change. There was a 1.4-fold decrease in geometric mean (GM) eNO (95% CI for difference 1.2-1.6-fold, *P *< 0.001), and no significant change in BHR.

**Table 3:** Change in Spirometry, eNO, and BHR Between 2 Visits in the Whole Population and for Atopic and Nonatopic Subjects Only*

		FEV_1 _(L)	FEV_1 _(% Predicted)	FVC (L)	FVC (% Predicted)	eNO (ppb)	PD_20 _(Doubling Doses)
All subjects	N	163	162	137	137	118	85
	Mean	-0.00	2.27†	0.02	2.33	-3.25†	0.29
	(95% CI)	(-0.06 to 0.06)	(0.29-4.26)	(-0.07 to 0.12)	(-0.50 to 5.17)	(1.22-1.62)	(-0.13 to 0.72)
Atopic	N	134	133	110	110	97	72
	Mean	-0.00	2.72†	0.04	3.30†	-3.93†	0.39
Nonatopic	(95% CI)	(-0.06 to 0.05)	(0.54-4.90)	(-0.06 to 0.14)	(0.18-6.42)	(1.25-1.73)	(-0.04 to 0.83)
	N	29	29	27	27	21	13
	Mean	-0.00	0.21	-0.06	-1.61	-1.10	-0.27
	(95% CI)	(-0.18 to 0.17)	(-4.83 to 5.24)	(-0.32 to 0.21)	(-8.57 to 5.35)	(0.86-1.61)	(-1.84 to 1.30)

*Lung function and BHR: mean changes (95% CI). eNO: geometric mean (95% CI for ratio).

†*P *< 0.05 vs baseline.

### Allergen Sensitization and Change in Lung Function, eNO, and BHR

Among atopic subjects, mean FEV_1 _improved from 82.1 to 84.8% predicted (95% CI for difference 0.5-4.9% predicted, *P *= 0.015) and FVC from 96.2 to 99.5% predicted (95% CI for difference 0.2-6.4% predicted, *P *= 0.038), although the absolute FEV_1 _and FVC did not change (Table 2). ENO decreased 1.5-fold (95% CI for difference 1.2-1.7-fold, *P *< 0.001). No such changes were observed in the nonatopic group.

### Role of Allergen Exposure

Subjects were classified as being exposed to high levels of domestic allergens based on previously proposed criteria (> 2 *μ*g/g Der p 1, > 10 *μ*g/g Fel d 1, and > 10 *μ*g/g Can f 1 in either carpet or mattress). Exposure to cat allergen was not associated with any changes in lung function, eNO, or BHR (Table 4).

**Table 4:** Change in Lung Function, eNO, and BHR Between 2 Visits According to Living Room Cat Allergen Exposure*

Fel d 1 (Living Room)		FEV_1 _(L)	FEV_1 _(% Predicted)	FVC (L)	FVC (% Predicted)	eNO (ppb)	PD_20 _(Doubling Doses)	Between Group Doubling Dose Difference PD_20 _(95% CI)
Not exposed	N	115	114	95	95	81	60	0.24
	Mean	0.00	1.93	0.03	2.18	-3.09†	0.33	(-0.70 to 1.17)
	(95% CI)	(-0.07 to 0.06)	(-0.48 to 4.35)	(-0.08 to 0.14)	(-1.26 to 5.62)	(1.14-1.68)	(-0.15 to 0.81)	
Exposed	N	49	49	42	42	38	26	*t *test, *P *= 0.615
	Mean	-0.03	2.68	0.01	2.67	-3.58†	0.10	ANOVA, *P *= 0.984
	(95% CI)	(-0.15 to 0.09)	(-0.92 to 6.28)	(-0.17 to 0.19)	(-2.55 to 7.90)	(1.20-1.75)	(-0.82 to 1.02)	

*Lung function and BHR: mean changes (95% CI). eNO: geometric mean (95% CI for ratio).

†*P *< 0.05 vs baseline.

We found no difference in the change in lung function or eNO between subjects exposed or not exposed to the high level of dust mite allergen or dog allergen, although those not exposed to dog allergen did show an increase in FEV_1 _over baseline of 2.4% predicted that was not seen in the dog-exposed group (Tables 5 and 6). BHR improved among subjects who were not exposed to high Der p 1 levels from GM PD_20 _= 359 *μ*g to GM PD_20 _= 632 *μ*g; no such improvement was observed in the exposed group (GM PD_20 _= 444 *μ*g at baseline vs GM PD_20 _= 327 *μ*g at follow-up), and there was a 1.26 doubling dose (DD) difference in the change versus baseline comparing the nonexposed group to the exposed group (95% CI for difference 0.44-2.08 DD, *P *= 0.003). In the multivariate ANOVA model adjusting for the potential confounders (age, inhaled steroid use and dose, smoking status, baseline PD_20_, sensitization to house dust mite, and exposure to high levels of dog allergen), high exposure to dust mite allergen remained significantly and independently associated with the change in BHR [estimated marginal means (95% CI): exposed -0.42 (-0.94 to 0.10) DD; nonexposed 0.86 (0.40-1.31) DD; *P *= 0.001 for difference; Table 5].

**Table 5:** Change in Lung Function, eNO, and BHR Between 2 Visits According to Mattress Exposure to Dust Mite Allergen*

Der p 1 (Mattress)		FEV_1 _(L)	FEV_1 _(% Predicted)	FVC (L)	FVC (% Predicted)	eNO (ppb)	PD_20 _(Doubling Doses)	Between Group Doubling Dose Difference PD_20 _(95% CI)
Not exposed	N	84	84	67	67	58	48	1.26
	Mean	-0.03	1.77	0.01	1.73	-2.89†	0.82†‡	(0.44 to 2.08)
	(95% CI)	(-0.12 to 0.05)	(-1.14 to 4.69)	(-0.13 to 0.15)	(-2.49 to 5.95)	(1.16-1.59)	(0.27-1.36)	
Exposed	N	80	79	70	70	61	38	*t *test, *P *= 0.003
	Mean	0.01	2.57	0.04	2.91	-3.60†	-0.44	ANOVA, *P *= 0.001
	(95% CI)	(-0.06 to 0.08)	(-0.17 to 5.31)	(-0.09 to 0.17)	(-1.00 to 6.82)	(1.14-1.85)	(-1.07 to 0.19)	

*Lung function and BHR: mean changes (95% CI). eNO: geometric mean (95% CI for ratio).

†*P *< 0.05 vs baseline; ‡*P *< 0.05 vs exposed.

**Table 6:** Change in Lung Function, eNO, and BHR Between 2 Visits According to Living Room Exposure to Dog Allergen*

Can f 1 (Living Room)		FEV_1 _(L)	FEV_1 _(% Predicted)	FVC (L)	FVC (% Predicted)	eNO (ppb)	PD_20 _(Doubling Doses)	Between Group Doubling Dose Difference PD_20 _(95% CI)
Not exposed	N	125	124	103	103	84	72	-1.00
	Mean	0.01	2.43†	0.04	2.51	-3.39†	0.10	(-2.14 to 0.14)
	(95% CI)	(-0.06 to 0.07)	(0.36-4.50)	(-0.07 to 0.14)	(-0.50 to 5.51)	(1.20-1.68)	(-0.39 to 0.58)	
Exposed	N	39	39	34	34	35	14	*t *test, *P *= 0.085
	Mean	-0.06	1.31	-0.01	1.81	-2.92†	1.10†	ANOVA, *P *= 0.019
	(95% CI)	(-0.19 to 0.07)	(-3.93 to 6.55)	(-0.24 to 0.22)	(-5.44 to 9.06)	(1.03-1.85)	(0.33-1.86)	

*Lung function and BHR: mean changes (95% CI). eNO: geometric mean (95% CI for ratio).

†*P *< 0.05 vs baseline.

In subjects exposed to high Can f 1 level, BHR improved from GM PD_20 _= 394 *μ*g to GM PD_20 _= 843 *μ*g over a period of 4 years, or a mean 1.10 DD improvement (95% CI for difference 0.39-1.80 DD, *P *= 0.005). This improvement was not seen in the nonexposed group (baseline GM PD_20 _= 394 *μ*g vs GM PD_20 _= 422 *μ*g at 4 years). There was a 1.01 DD difference in change versus baseline comparing the exposed group to the nonexposed group (95% CI for difference -0.01 to 2.12 DD, *P *= 0.07). In the multivariate ANOVA model adjusting for the potential confounders (age, inhaled steroid use and dose, smoking status, exposure to house dust mite allergen, and baseline PD_20_), high exposure to dog allergen was significantly and independently associated with the improvement in BHR [estimated marginal means (95% CI): exposed 1.17 (0.35-1.99) DD; nonexposed (-0.28 to 0.45) DD; *P *= 0. 019 for difference; Table 6].

To minimize the possibility that a change in dog ownership status may have confounded the results, the ANOVA model was repeated, first including "lost a dog between visits 1 and 2" as a confounder and second excluding from the analysis those subjects who had lost their dog. There was no change in the results in either case. For example, when subjects who got rid of their dog between visits 1 and 2 were excluded, there was again no change in BHR for those not exposed to high levels of dog allergen [n = 70, mean (95% CI) 0.11 (-0.39 to 0.60) DD change] but BHR decreased in those exposed to high Can f 1 levels [n = 11, mean (95% CI) (0.20-1.99) DD change, *P *= 0.022]. High dog allergen exposure was again significantly associated with the change in BHR in the multivariate ANOVA model (confounders: age, number of years with asthma, inhaled steroid use and dose, smoking status, dog sensitization, exposure to house dust mite allergen, and baseline PD_20_; *P *= 0.04).

**Figure 1:**
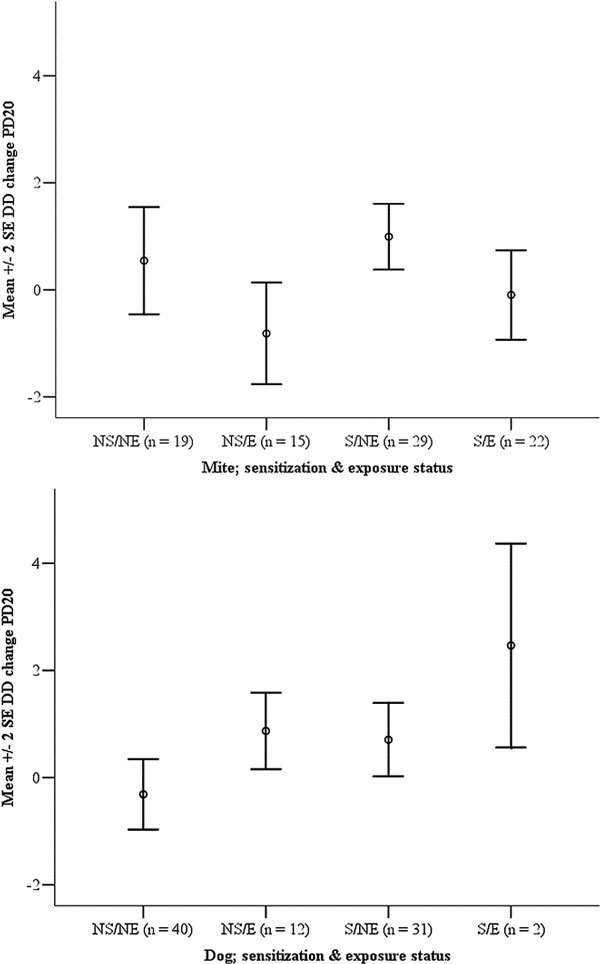
**Mean (± 2 SE) PD_20 _as doubling dose change from baseline by sensitization and exposure to mite (top) and dog (bottom)**. NS, not sensitized; NE, not exposed; S, sensitized; E, exposed.

For each allergen there was no significant effect of the interaction between exposure and sensitization in the multivariate models (data not shown). This was indirectly confirmed when we carried out the analysis according to the exposure and sensitization status (not sensitized/not exposed, not sensitized/exposed, sensitized/not exposed, and sensitized/exposed; Figure 1).

**Figure 2:**
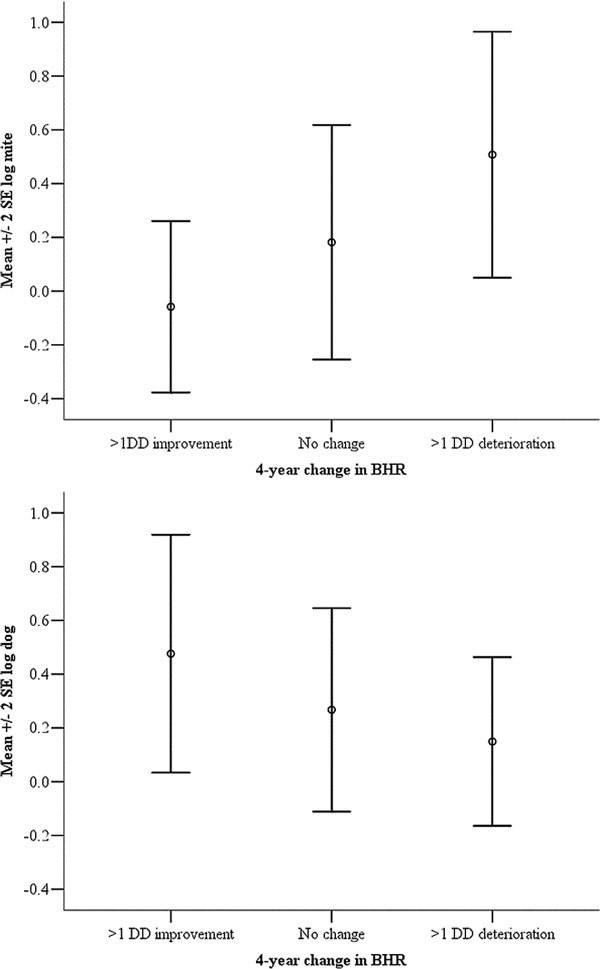
**Mean (± 2 SE) log allergen levels for mattress mite (top) and living room dog (bottom) categorized by doubling dose change in PD_20_**.

We then compared the actual allergen levels between individuals with > 1 DD increase in PD_20 _(deterioration), > 1 DD decrease in PD_20 _(improvement), and no change in BHR (the difference within 1 DD between 2 visits). Der p 1 decreased significantly, whereas Can f 1 increased significantly with the improving BHR (Figure 2). Subjects with > 1 DD deterioration in PD_20 _or more had 2.9-fold higher levels of Der p 1 detected in their mattress compared with those with no change or an improvement in PD_20 _(95% CI for difference 0.87-9.31 *μ*g/g; multivariate ANOVA model, *P *= 0.046). For dog allergen, subjects with > 1 DD improvement in PD_20 _were exposed to 2.7-fold more dog allergen compared with those with no improvement or a deterioration in PD_20 _(95% CI for difference 0.07-2.81 *μ*g/g; multivariate ANOVA model, *P *= 0.04).

## Discussion

### Principal Findings

We have shown that exposure to high levels of dust mite allergen in persons with asthma was associated with a deterioration in BHR over a period of 4 years. The converse was true for dog allergen in that high exposure was associated with an improvement in BHR. These findings were in a large group of well-characterized persons with asthma and remained significant after potential confounders were introduced to the model. There was no independent effect of sensitization to the relevant allergen in this model.

### Limitations

We are aware of the possible selection bias in our data set. Only a proportion of the original attendees were reviewed after 4 years, and those who agreed to attend for a second visit may not be representative of the whole cohort. Although we obtained domestic dust samples from more than 80% of these subjects, one of the primary end points (change in BHR) was measured at both visits in only 86 subjects. As we have acknowledged, these were not entirely typical of the group as a whole, in general, having milder disease requiring less treatment and with better lung function. This is in part because (for the reasons of safety) we did not carry out bronchial challenge in patients with marked airway obstruction (FEV_1 _< 60% predicted), and thus more severe asthma.

Another important potential confounder in our study is the high proportion of subjects who were taking inhaled steroids, at a reasonably high mean dose. This may well account for the mean improvements seen overall in eNO and FEV_1 _percentage predicted over the study period. We included inhaled steroid use and dose as a covariate in the multivariate models where appropriate for this reason. We used a skin prick test wheal of minimum 3 mm greater than control to determine atopy. It is possible therefore that we underestimated sensitization, and future studies should include measurements of specific radioallergosorbent test IgE to minimize this possibility.

Regarding the unexpected findings with dog allergen exposure, there is a chance of type II error with only 14 subjects exposed. This may of course represent self-selection, with persons having more severe asthma avoiding dog exposure in the home, although there were no differences in other markers of asthma severity, such as PD_20_, eNO, FEV_1_, or inhaled corticosteroid dose at baseline between groups according to dog exposure (data not shown). It is also possible that domestic allergen exposure changed over the 4-year period, although a recent study has demonstrated acceptable stability over a similar period,[20] at least for mite and cat allergens.

### Meaning of the Study

That exposure to high levels of house dust mite allergen was associated with deteriorating BHR was not an unexpected finding. House dust mite allergens include a number of proteases and trypsins that may disrupt cell membranes and have direct pro-inflammatory actions, and they also mediate airway inflammation through IgE activation [21]. They may therefore contribute to the persistence of airway inflammation over time, and perhaps a consequent increase in bronchial hyperresponsiveness.

Early-life exposure to dogs is associated with a reduced likelihood of developing asthma in children,[22] but there are no data for adults with asthma. It is interesting therefore that we have found an association between exposure to high levels of dog allergen and improvement of BHR. We considered the possibility that subjects may have gotten rid of their dog between visits, but even after controlling for this, we discovered that the association remained the same. It is also possible that dog ownership per se conferred the observed improvements. However, when we tested for an association between dog ownership and change in BHR, we found none (data not shown). One other possibility is that tolerance resulting from high dog allergen exposure may have developed in the skin prick negative subjects and could be responsible for some of the difference seen. Although there was no correlation between levels of dust mite and dog allergens (data not shown), it is still possible of course that there are other important confounding variables that we have not measured, such as domestic levels of endotoxin or fungal spores. The relationship between exposure to endotoxin or mold and asthma is unclear. Some studies have shown an inverse association with allergic sensitization and asthma in children,[23,24] but others demonstrated increased risk for asthma prevalence [25].

The majority of our subjects were atopic, and this group as a whole improved over time in terms of lung function and eNO, whereas the nonatopic group did not. Dijkstra and colleagues followed 122 persons with asthma for more than 23 years and reported no association between atopy and lung function change [26]. However, atopy was more common (98% vs 87%) in the 159 subjects excluded from the analysis, either because they had never used inhaled corticosteroids or had insufficient longitudinal data. It is likely, therefore, that this group had less severe asthma. Another longitudinal study reported a lower prevalence of irreversible airflow obstruction in persons with atopic asthma [6]. It is likely that different, although not necessarily independent, factors are important when considering asthma development, asthma severity, and asthma progression. It is also likely that these factors may differ between recognized asthma phenotypes, such as late onset versus early onset or atopic versus nonatopic.

## Conclusions

We have demonstrated that exposure to high levels of house dust mite allergen in the home, over a period of 4 years, is associated with deteriorating bronchial hyperresponsiveness, whereas the converse is true for dog allergen exposure. If these findings are confirmed over longer periods of time, further investigation into the favorable association between dog allergen exposure and prognosis will be required, and possibly targeted intervention in those exposed to high levels of house dust mite allergen, irrespective of their sensitization status.

## Note

Supported by an endowment fund from South Manchester University Hospitals NHS Trust.

Presented at the 2007 AAAAI Annual Meeting, February 23-27, 2007, San Diego.

## References

[B1] Lange P Parner J Vestbo J Schnohr P Jensen G A 15-year follow-up study of ventilatory function in adults with asthma N Engl J Med 1998 2 1194 1200 10.1056/NEJM199810223391703 9780339

[B2] Masoli M Fabian D Holt S Beasley R Global Initiative for Asthma (GINA) Program The global burden of asthma: executive summary of the GINA Dissemination Committee report Allergy 2004 2 469 478 10.1111/j.1398-9995.2004.00526.x 15080825

[B3] Taylor RG Joyce H Gross E Holland F Pride NB Bronchial reactivity to inhaled histamine and annual rate of decline in FEV1 in male smokers and ex-smokers Thorax 1985 2 9 16 10.1136/thx.40.1.9 3969664 PMC459970

[B4] Barter CE Campbell AH Relationship of constitutional factors and cigarette smoking to decrease in 1-second forced expiratory volume Am Rev Respir Dis 1976 2 305 314 1259240 10.1164/arrd.1976.113.3.305

[B5] Grol MH Gerritsen J Vonk JM Schouten JP Koeter GH Rijcken B Postma DS Risk factors for growth and decline of lung function in asthmatic individuals up to age 42 years A 30-year follow-up study Am J Respir Crit Care Med 1999 2 1830 1837 10.1164/ajrccm.160.6.9812100 10588593

[B6] Vonk JM Jongepier H Panhuysen CI Schouten JP Bleecker ER Postma DS Risk factors associated with the presence of irreversible airflow limitation and reduced transfer coefficient in patients with asthma after 26 years of follow up Thorax 2003 2 322 327 10.1136/thorax.58.4.322 12668795 PMC1746641

[B7] Simpson A Soderstrom L Ahlstedt S Murray CS Woodcock A Custovic A IgE antibody quantification and the probability of wheeze in preschool children J Allergy Clin Immunol 2005 2 744 749 10.1016/j.jaci.2005.06.032 16210045

[B8] Fowler SJ Lipworth BJ Relationship of skin-prick reactivity to aeroallergens and hyperresponsiveness to challenges with methacholine and adenosine monophosphate Allergy 2003 2 46 52 10.1034/j.1398-9995.2003.23779.x 12580806

[B9] Gore RB Curbishley L Truman N Hadley E Woodcock A Langley SJ Custovic A Intranasal air sampling in homes: relationships among reservoir allergen concentrations and asthma severity J Allergy Clin Immunol 2006 2 649 655 10.1016/j.jaci.2005.12.1351 16522466

[B10] Langley SJ Goldthorpe S Craven M Woodcock A Custovic A Relationship between exposure to domestic allergens and bronchial hyperresponsiveness in non-sensitised, atopic asthmatic subjects Thorax 2005 2 17 21 10.1136/thx.2004.027839 15618577 PMC1747172

[B11] Langley SJ Goldthorpe S Craven M Morris J Woodcock A Custovic A Exposure and sensitization to indoor allergens: association with lung function, bronchial reactivity, and exhaled nitric oxide measures in asthma J Allergy Clin Immunol 2003 2 362 368 10.1067/mai.2003.1654 12897743

[B12] Kharitonov S Alving K Barnes PJ Exhaled and nasal nitric oxide measurements: recommendations. The European Respiratory Society Task Force Eur Respir J 1997 2 1683 1693 10.1183/09031936.97.10071683 9230267

[B13] Standardization of Spirometry, 1994 Update. American Thoracic Society Am J Respir Crit Care Med 1995 2 1107 1136 7663792 10.1164/ajrccm.152.3.7663792

[B14] Juniper EF Frith PA Dunnett C Cockcroft DW Hargreave FE Reproducibility and comparison of responses to inhaled histamine and methacholine Thorax 1978 2 705 710 10.1136/thx.33.6.705 746496 PMC470967

[B15] Salome CM Schoeffel RE Woolcock AJ Comparison of bronchial reactivity to histamine and methacholine in asthmatics Clin Allergy 1980 2 541 546 10.1111/j.1365-2222.1980.tb02135.x 7438414

[B16] Yan K Salome C Woolcock AJ Rapid method for measurement of bronchial responsiveness Thorax 1983 2 760 765 10.1136/thx.38.10.760 6648855 PMC459653

[B17] Luczynska CM Arruda LK Platts-Mills TA Miller JD Lopez M Chapman MD A two-site monoclonal antibody ELISA for the quantification of the major Dermatophagoides spp. allergens, Der p I and Der f I J Immunol Methods 1989 2 227 235 10.1016/0022-1759(89)90010-0 2926155

[B18] Chapman MD Aalberse RC Brown MJ Platts-Mills TA Monoclonal antibodies to the major feline allergen Fel d I. II Single step affinity purification of Fel d I, N-terminal sequence analysis, and development of a sensitive two-site immunoassay to assess Fel d I exposure J Immunol 1988 2 812 818 3276780

[B19] Schou C Hansen GN Lintner T Lowenstein H Assay for the major dog allergen, Can f I: investigation of house dust samples and commercial dog extracts J Allergy Clin Immunol 1991 2 847 853 10.1016/0091-6749(91)90240-O 1744355

[B20] Antens CJ Oldenwening M Wolse A Gehring U Smit HA Repeated measurements of mite and pet allergen levels in house dust over a time period of 8 years Clin Exp Allergy 2006 2 1525 1531 10.1111/j.1365-2222.2006.02603.x 17177675

[B21] Asokananthan N Graham PT Stewart DJ Bakker AJ Eidne KA Thompson PJ Stewart GA House dust mite allergens induce proinflammatory cytokines from respiratory epithelial cells: the cysteine protease allergen, Der p 1, activates protease-activated receptor (PAR)-2 and inactivates PAR-1 J Immunol 2002 2 4572 4578 12370395 10.4049/jimmunol.169.8.4572

[B22] Waser M von Mutius E Riedler J Nowak D Maisch S Exposure to pets, and the association with hay fever, asthma, and atopic sensitization in rural children Allergy 2005 2 177 184 10.1111/j.1398-9995.2004.00645.x 15647038

[B23] Gehring U Bischof W Fahlbusch B Wichmann HE Heinrich J House dust endotoxin and allergic sensitization in children Am J Respir Crit Care Med 2002 2 939 944 10.1164/rccm.200203-256OC 12359650

[B24] Braun-Fahrlander C Riedler J Herz U Eder W Waser M Environmental exposure to endotoxin and its relation to asthma in school-age children N Engl J Med 2002 2 869 877 10.1056/NEJMoa020057 12239255

[B25] Thorne PS Kulhankova K Yin M Cohn R Arbes SJ Jr Zeldin DC Endotoxin exposure is a risk factor for asthma: the national survey of endotoxin in United States housing Am J Respir Crit Care Med 2005 2 1371 1377 10.1164/rccm.200505-758OC 16141442 PMC1379232

[B26] Dijkstra A Vonk JM Jongepier H Koppelman GH Schouten JP ten Hacken NH Lung function decline in asthma: association with inhaled corticosteroids, smoking and sex Thorax 2006 2 105 110 10.1136/thx.2004.039271 16308336 PMC2104585

